# Primary Small Cell Cancer of the Breast: An Unusual Presentation

**DOI:** 10.7759/cureus.81710

**Published:** 2025-04-04

**Authors:** Jay K Garlapati, Desten Howard, Jordan Maier, Michael C Joiner, Steven R Miller

**Affiliations:** 1 Department of Oncology, Wayne State University School of Medicine, Detroit, USA; 2 Department of Pathology, Wayne State University School of Medicine, Detroit, USA

**Keywords:** external radiation therapy, intensive chemotherapy, neuroendocrine carcinoma (nec), rare breast cancer, small cell carcinomas

## Abstract

Primary small cell carcinoma of the breast (PSCCB) is a rare but aggressive disease with multiple treatment options available, including surgery, chemotherapy, and radiation therapy. We report a case of a 51-year-old female patient who presented with a palpable left breast mass. An ultrasound-guided core needle biopsy of the affected area revealed triple-negative small cell carcinoma of the left breast. The patient underwent neoadjuvant chemotherapy, breast-conserving surgery with negative margins, and post-operative radiation therapy. One year later, the patient developed pathologic evidence of metastatic disease. Despite receiving three additional chemotherapeutic regimens and site-specific radiation treatments, the patient continued to have widespread metastatic disease. This case underscores the challenges of diagnosing and treating PSCCB and the urgent need for creating a standardized treatment protocol to improve patient outcomes.

## Introduction

Primary small cell carcinoma of the breast (PSCCB) is an exceptionally rare and highly aggressive malignancy, accounting for less than one percent of all breast cancers. Over half of all newly diagnosed patients with PSCCB present with nodal involvement, a unique characteristic that sets it apart from other breast cancers. In comparison, triple-negative breast cancer (estrogen (ER), progesterone (PR), and human epidermal growth factor receptor 2 (HER2) receptor negative) accounts for 10-20% of all breast cancers and is more prevalent [1,2]. The exact etiology of PSCCB is unknown and likely multifactorial, but most affected patients are women over 60. The World Health Organization's 2019 (fifth edition) breast cancer classification categorizes breast carcinomas with neuroendocrine features into three groups: well-differentiated neuroendocrine tumors, which architecturally resemble carcinoid tumors of other sites; moderately differentiated (atypical carcinoids); and poorly differentiated [3].

Diagnosing PSCCB requires the clinical exclusion of metastasis from a non-mammary primary site [4]. Immunohistochemical workup can aid in this distinction as tumor cells from PSCCB display recognizable differences compared to small cell carcinomas originating from a non-mammary organ. Often, they maintain recognizable features of the primary tumor. Most available information about this rare disease comes from case reports and retrospective studies. Many of these studies have reported treating PSCCB with a multimodal approach involving surgery, chemotherapy, external beam radiation therapy (EBRT), and hormonal therapy. Of note is that the chemotherapeutic agents administered to patients in these reports are treatments commonly utilized for small-cell lung cancer.

We present a case of a 51-year-old female patient diagnosed with PSCCB who, despite undergoing breast-conserving surgery, EBRT, multiple chemotherapy regimens, and various site-specific radiation treatments, developed metastatic disease. This case underscores the urgent need for creating a standardized treatment protocol for PSCCB and reviews the current therapeutic strategies for this rare disease.

## Case presentation

**Figure 1:**
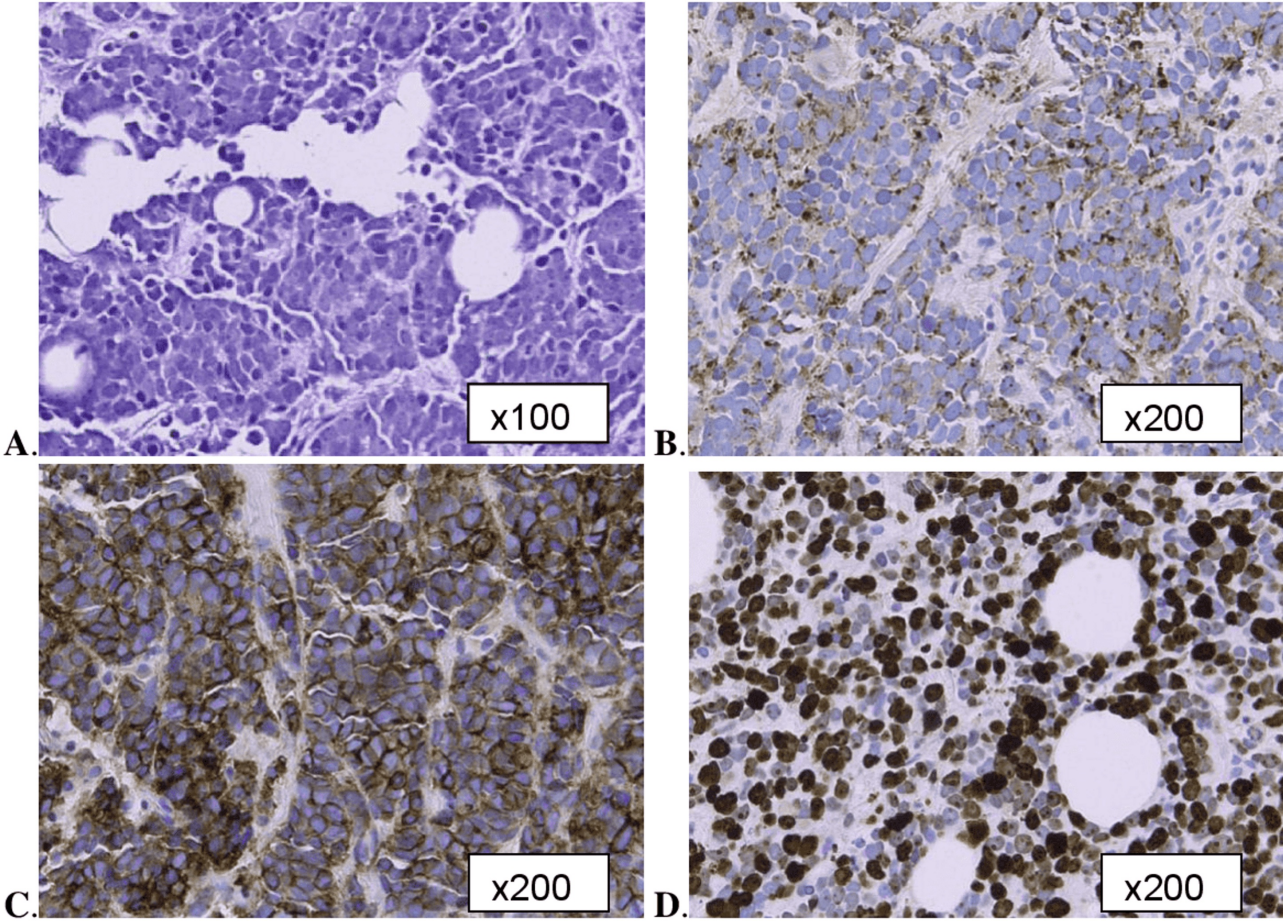
The tumor shows nests of cells with intervening fibrous tissue. The cells are monotonous with indistinct borders, nuclear molding, scant cytoplasm and brisk mitoses (A). The nuclei are hypochromatic and round to oval with fine, granular chromatin and prominent nucleoli. Immunohistochemical staining is patchy perinuclear for CAM 5.2 (cytokeratin CAM 5.2, which is a low-molecular-weight keratin that primarily reacts with human keratin proteins) (B) and has diffuse membranous positivity for CD56 (C). The Ki-67 index (a marker for cell proliferation) is >85% (D)

A postmenopausal woman in her 50s with a past medical history of tuberculosis treated over 20 years ago noticed a mass in her left breast during a self-breast exam. Four months later, a routine mammogram identified a 4-cm mass in the left breast and an enlarged 1.4-cm left axillary lymph node. The initial differential diagnosis included benign conditions such as fibroadenoma, fat necrosis, and tuberculosis mastitis, as well as malignant etiologies. An ultrasound-guided biopsy was performed of both the left breast mass and the lymph node, and pathology was consistent with a small cell carcinoma involving the left breast and the axillary lymph node. Immunohistochemical stains were negative for ER, PR, HER2, and GATA binding protein 3 receptor activity. (GATA3 is a transcription factor encoded by the GATA3 gene in humans and controls the expression of a wide range of biologically and clinically significant genes.) [5] The morphological features of nests/clusters of small cells with nuclear molding, fine/granular chromatin, and scant cytoplasm with thin intervening fibrous stroma were noted to be features consistent with a small cell tumor. Staining for AE1/AE3, which distinguishes carcinomas from other tumors, such as sarcomas or lymphomas, was positive [6], and chromogranin and synaptophysin stains were negative, which helped to rule out neuroendocrine differentiation, thus supporting the diagnosis of small cell carcinoma. The CD56 stain was positive, an immunohistochemical marker used in anatomic pathology to recognize certain tumors and help support the diagnosis of small cell carcinoma (Figure 1) [7].

**Figure 2:**
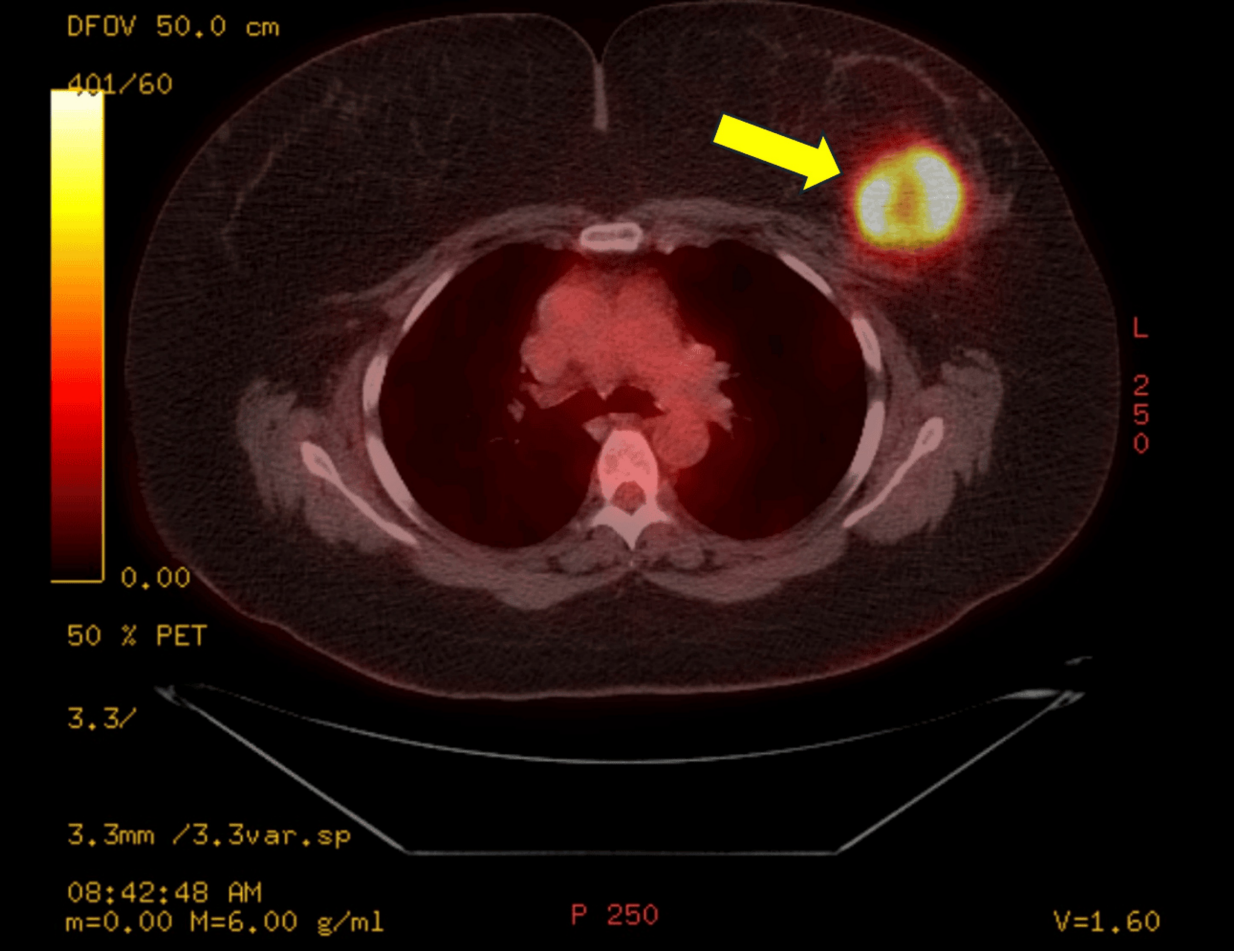
Positron emission tomography (PET) with the yellow arrow pointing to the lesion involving the left breast

Staging studies including a positron emission tomography (PET) scan were obtained which identified a 5.3 cm necrotic mass in the left breast. No evidence of metastatic disease was noted (Figure 2).

**Figure 3:**
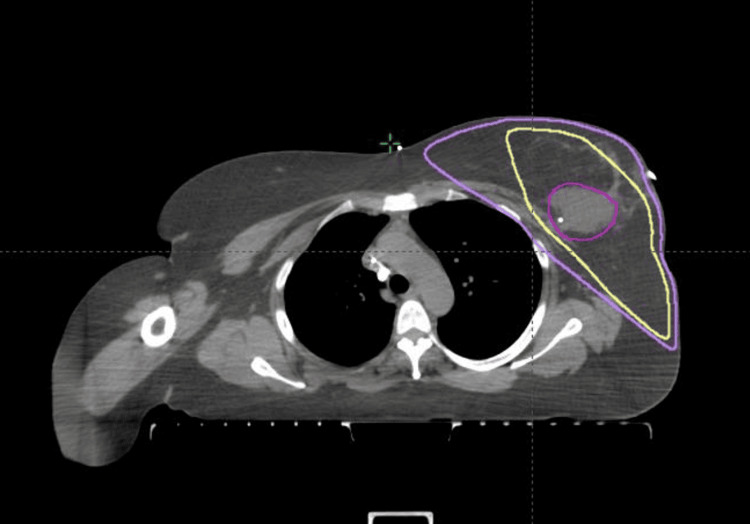
Axial view of the isodose lines for the left breast and tumor bed EBRT. Yellow is the 5272 cGy isodose line, purple is the 4272 cGy isodose line covering the whole left breast, and the pink contour is the lumpectomy cavity EBRT: External beam radiation therapy

After the completion of initial imaging studies, the patient decided to proceed with breast-conserving therapy, lumpectomy, and adjuvant left breast EBRT. She initially underwent neoadjuvant chemotherapy in an effort to downsize the breast primarily and to aid with breast-conserving treatment as well as to treat possible metastatic disease. She received six cycles of neoadjuvant carboplatin and etoposide, followed by a lumpectomy, which resulted in the removal of a 1.7 cm tumor from the left breast with negative margins. In addition, one axillary lymph node was removed, which was negative for malignancy. She subsequently underwent four weeks of EBRT to the left breast. She was treated with a two-field technique consisting of a medial and lateral tangent field, treating the left breast to a dose of 4272 centigray (cGy) in 16 fractions of 267 cGy per fraction using a combination of 15 and 6 mV photons. The lumpectomy cavity with a 1 cm margin was treated to an additional 1000 cGy in 4 fractions of 250 Gy fractions using 15 mV photons (Figure 3).

**Figure 4:**
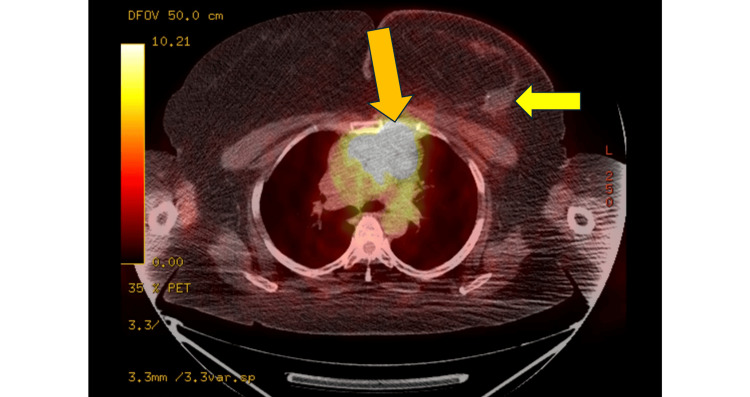
PET scan displaying mediastinal disease (orange arrow). Also note that the left breast tumor bed is negative on the PET scan (yellow arrow)

One year after completing her neoadjuvant chemotherapy, surgery, and adjuvant left breast EBRT, a surveillance PET scan demonstrated increased avidity in the left supraclavicular, infraclavicular, and mediastinal lymph nodes. A CT-guided mediastinal lymph node biopsy was obtained, and pathology was consistent with a poorly differentiated carcinoma with non-specific glandular differentiation. (It remains unclear if this signified a possible recurrence from the poorly differentiated adenocarcinoma of the left breast or a de novo non-small cell lung cancer.) Of note, there was no evidence of disease in bilateral breasts or axilla (Figure 4).

**Figure 5:**
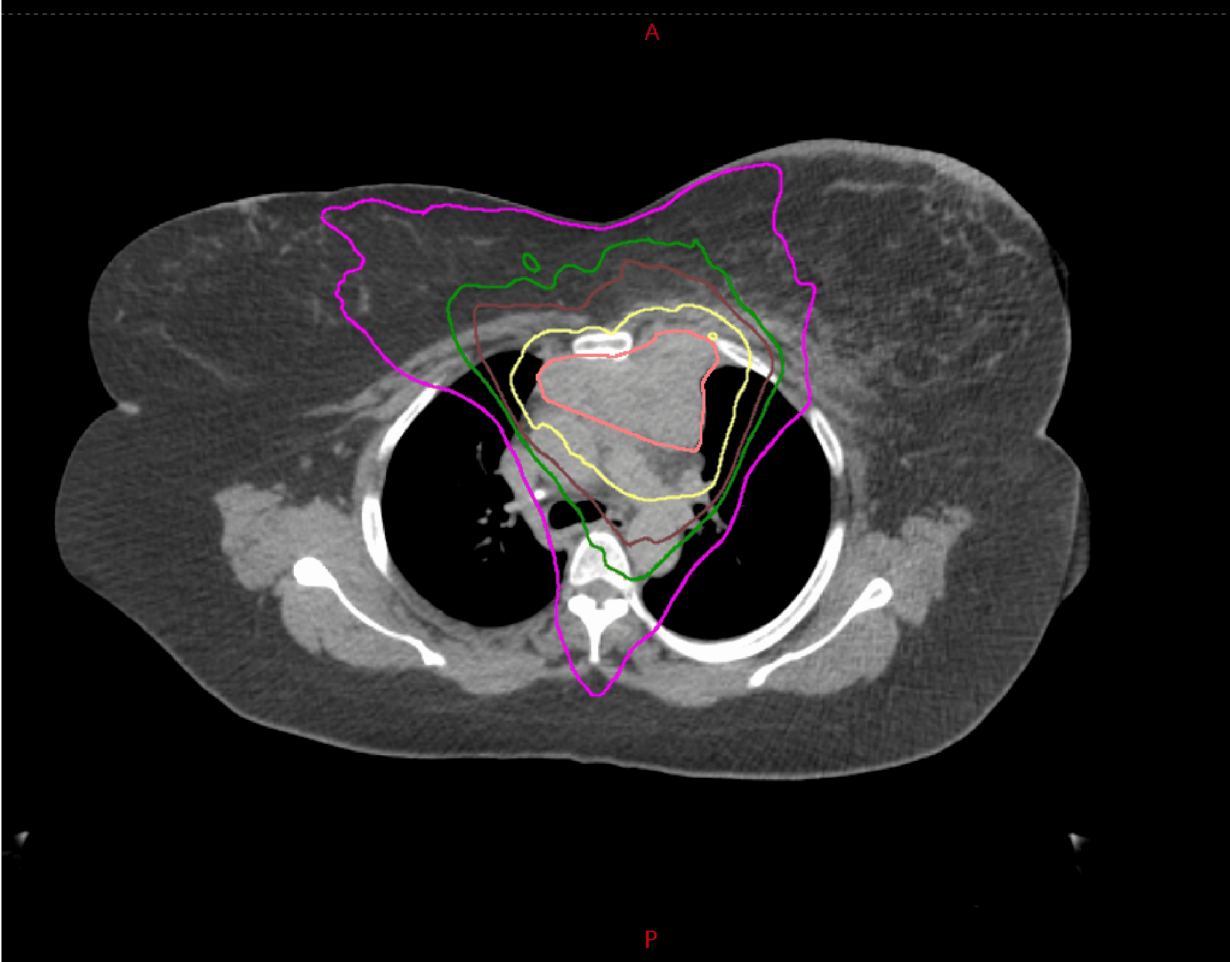
Radiation treatment of the mediastinal disease. Purple is the 3000 cGy isodose line, dark green is the 4200 cGy isodose line, brown is the 4800 cGy isodose line, yellow is the 6000 cGy isodose line, and peach is the mediastinal tumor seen on the PET scan

She subsequently underwent concurrent chemotherapy (paclitaxel and carboplatin) and EBRT. The gross tumor volume (GTV) was the PET-positive disease, and the planning target volume (PTV) was an additional 5 mm margin around the GTV. Intensity-modulated radiation therapy using 6 mV photons was utilized to administer a dose of 6000 cGy in 30 fractions to the PTV (Figure 5). 

Mild esophagitis was a notable acute side effect, but the patient did not experience any long-lasting adverse treatment outcomes. After treatment completion, one year of immunotherapy was recommended, but the patient pursued surveillance instead.

**Figure 6:**
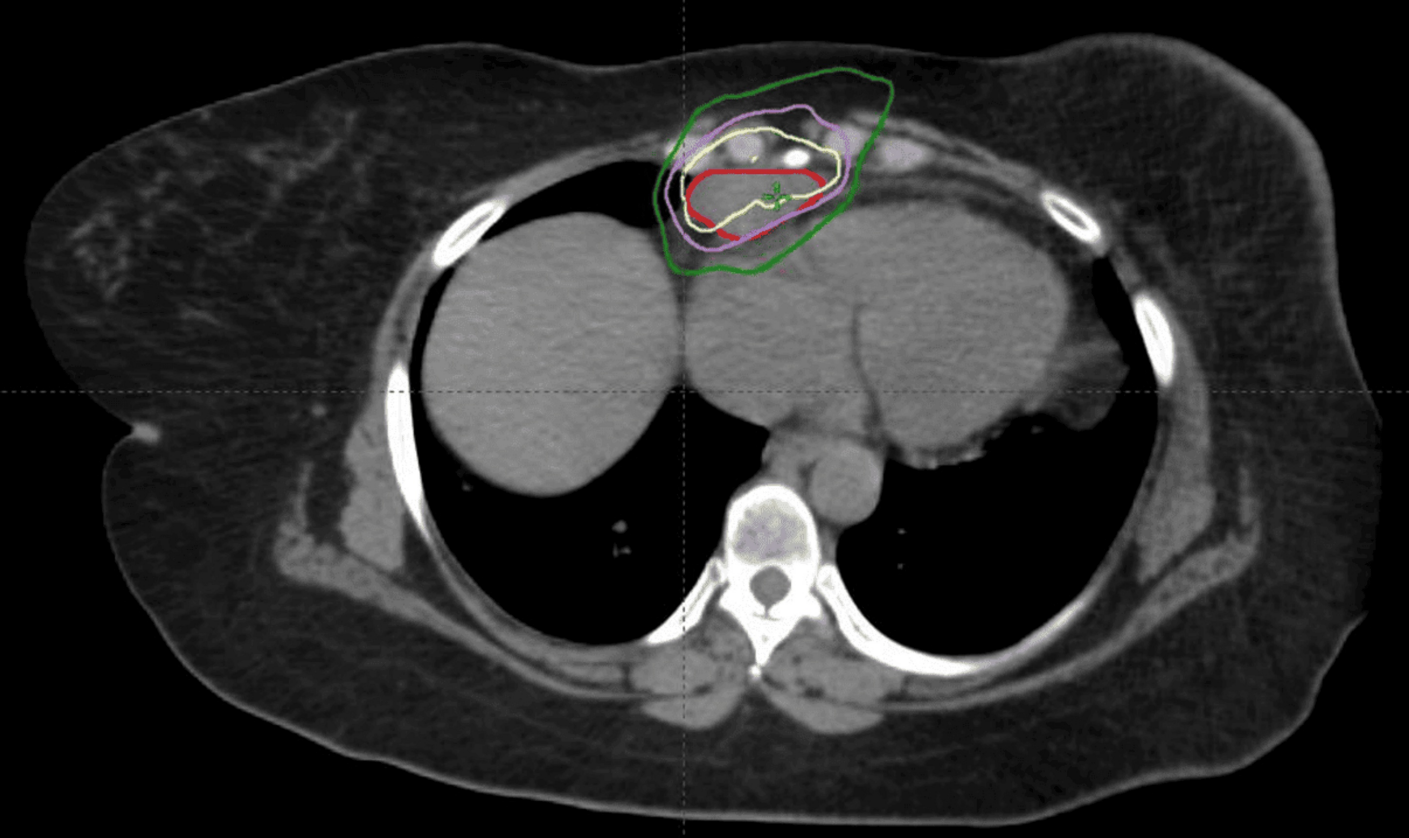
Radiation doses to the costophrenic lymph node. Dark red is the GTV, yellow is the 100% isodose line, purple is the 90% isodose line, and green is the 70% isodose line GTV: Gross tumor volume

Fifteen months after the completion of her radiation to the mediastinal disease, a routine CT scan of the chest identified a new right cardiophrenic lymph node, which was proven to be avid by a subsequent PET scan. A biopsy of this lymph node revealed small cell carcinoma, which likely metastasized from the left breast. The patient underwent stereotactic body radiation therapy (SBRT) to the affected lymph node to a total dose of 3000 cGy in 5 fractions utilizing 6 mV photons (Figure 6). The GTV was the PET positive disease, and the PTV was a 0.5 cm margin around the GTV in all dimensions, and SBRT was used to limit the radiation dose to the heart and lungs as well as minimize any overlap with her previous EBRT.

Three months after undergoing SBRT to the right cardiophrenic node, the patient presented to the emergency room with shortness of breath. A CT scan with contrast of the thorax, abdomen, and pelvis at that time revealed multiple nodules or masses in the mediastinum, anterior pericardium, peritoneum, and the right base of the neck along with lytic lesions in the lumbar spine and sacrum. 750 cc of fluid was removed during a right-sided thoracentesis, and cytology revealed malignant cells. As a result, the patient received four cycles of carboplatin, etoposide, and durvalumab, as well as maintenance durvalumab. (This decision was made based on the CASPIAN trial, which showed significant improvement in overall survival in patients with extensive stage small cell lung cancer who received durvalumab and platinum-etoposide agents [8].)

Five months later, the patient underwent palliative EBRT to the painful and edematous soft tissue mass in the base of her right neck. She received 3000 cGy in 10 fractions using three-dimensional treatment planning and 15 mV photons. Considerable improvement in the right upper extremity was noted.

A surveillance CT of the thorax and abdomen, approximately 1.5 years since the initiation of treatment, revealed new liver lesions and enlarged right cervical lymph nodes. Therefore, durvalumab was stopped, and lurbinectidin was initiated, which was poorly tolerated. Following the first cycle of lurbinectidin, the patient experienced significant nausea, diarrhea, fatigue, and severe cytopenia. As a result, the dose of lurbinectidin was reduced during the second cycle, which was better tolerated. She has recently completed two additional cycles of lurbinectidin interval imaging, which revealed stable disease.

## Discussion

Carcinoma of the breast is one of the most common malignancies affecting women, and atypical breast cancers comprise a vast minority of these cases. In particular, PSCCB constitutes less than one percent of all breast cancers [1]. Similarly, small-cell lung carcinomas most commonly appear in the lungs, but up to five percent can occur in extrapulmonary sites [9]. The limited information available concerning PSCCB is derived mainly from case reports. Many of these reports describe treating PSCCB by extrapolating therapeutic strategies from small-cell lung carcinoma, which aligns with the approach used for our patient. This is likely because PSCCB is morphologically and histologically similar to pulmonary small-cell carcinomas [10].

The exact etiology of PSCCB remains unknown; however, a population-based study comparing risk factors between 83 patients with PSCCB and 410,699 patients with invasive ductal carcinoma found that PSCCB was more commonly associated with older age, male sex, higher tumor stage, and grade, a higher proportion of triple-negative breast cancer, an increased likelihood of developing metastatic disease and worse outcomes [10].

A similar study by Zhu et al. included 323 patients with PSCCB and discovered the highest incidence is in women between the ages of 65 and 69, with worse outcomes in those with advanced disease. The study's five-year disease-specific survival rates for stage I and stage IV disease were 94.3% and 8.8%, respectively, and the five-year overall survival rates for stage I and stage IV disease were 85.7% and 4.6% [11]. In comparison, the five-year survival rate for localized and distant triple-negative breast cancer was 91% and 77%, respectively [12,13]. 

PSCCB is an aggressive disease with the potential for rapid progression. According to a review of 53 patients by Kanat et al., axillary node metastasis was present in 61.7% of cases, and tumor size ranged from 1 to 18 cm, with a mean size of 4.53 cm [2]. Additionally, immunohistochemical stains can assist in diagnosis, with chromogranin A and synaptophysin being the most sensitive and specific markers [12,14]. However, these markers are not always present and only support the diagnosis of PSCCB rather than exclude it [4,15].

As previously mentioned, no standard treatment protocol for PSCCB has been established. Case reports and retrospective studies offer insight into the effectiveness of a multimodal approach involving surgery, chemotherapy, and radiation therapy. Hormonal therapy can also be added if the tumor expresses appropriate hormone receptors [16]. From our review of the literature, most case reports and studies describe surgery as either a lumpectomy with negative margins or mastectomy of the affected breast(s) and adjuvant breast irradiation. In contrast, chemotherapeutic agents involved in treating PSCCB were discussed in greater detail and have shown considerable variability. Several articles reported the use of anthracycline- and taxane-based chemotherapy regimens, which are commonly employed for invasive breast cancer [11,17]. However, many other studies described platinum-based agents and etoposide, a regimen typically associated with small-cell lung cancer [2,15,17-21]. Notably, a few studies documented the combination of anthracycline- and taxane-based agents with platinum-based agents and etoposide [22-24]. Our patient was treated with platinum-based agents and etoposide since more data was needed to support treating PSCCB with this regimen.

In a retrospective study by Zhu et al., 73.7% of all patients diagnosed with stage I or II PSCCB typically underwent surgery; however, follow-up studies detailing their outcomes were lacking [11]. This study also claimed that chemotherapy regimens involving anthracycline- and taxane-based agents have a significant impact on survival and that radiation therapy is controversial. 

In contrast, Kanat et al. encountered that, out of 53 patients, 50.9% underwent a modified radical mastectomy, 69.8% underwent chemotherapy, and 39.6% of patients had adjuvant radiotherapy. Cisplatin and etoposide or 5-fluorouracil, epirubicin, and cyclophosphamide were the predominant chemotherapy regimens administered in this trial. With a mean follow-up of 20.75 months, 18.9% of the patients diagnosed with PSCCB died of metastatic disease. The prognosis of patients in this study appeared to strongly correlate with the initial stage of the disease [2].

In addition, Suhani et al. reviewed four patients with primary neuroendocrine carcinoma of the breast. All four patients were treated with modified radical mastectomy, six cycles of cyclophosphamide, adriamycin, and 5-fluorouracil-based adjuvant chemotherapy, radiotherapy, and hormonal therapy. With a mean follow-up time of 27.7 months (range 48-9), all four patients survived without loco-regional or metastatic recurrence [22].

The role of radiation therapy in treating PSCCB remains unclear. In a population-based study, Hare et al. analyzed 199 patients with PSCCB, 69 of whom received EBRT and 123 who did not. Their findings revealed no significant improvement in overall survival with EBRT. However, these patients were diagnosed and treated between 1973 and 2010, and the study lacked data on key variables such as tumor size, tumor grade, the type and dose of radiation used, and chemotherapy regimens, all of which may have impacted the results [25].

Based on the trials outlined above and numerous case reports, a multimodal approach of surgery, chemotherapy, radiation, and hormonal therapy is commonly employed. While these therapies demonstrate varying degrees of success, further research is needed to establish a standardized treatment protocol for PSCCB. Immunotherapy and targeted therapies could also play a role, but these treatments have not been well studied for PSCCB. An improved understanding of the molecular pathways involved in PSCCB could identify novel therapeutic agents such as antibody-drug conjugates and poly-ADP ribose polymerase inhibitors.

## Conclusions

Our case depicts the aggressive nature of PSCCB, evidenced by the rapid progression to metastatic disease in our patient. Although not formally tested in a randomized control trial, early diagnosis and treatment with multimodal therapy of PSCCB are likely to provide the best chance of survival. This should include an extensive histochemical workup, early involvement of the multidisciplinary tumor board, and careful supervision of the patient. Appropriate patient counseling regarding treatment adherence and follow-up is needed. Additionally, ongoing reporting of PSCCB is crucial for improving understanding, diagnosis, and treatment of this rare disease.
